# miR-34a and miR-200c Have an Additive Tumor-Suppressive Effect on Breast Cancer Cells and Patient Prognosis

**DOI:** 10.3390/genes12020267

**Published:** 2021-02-12

**Authors:** Behzad Mansoori, Nicola Silvestris, Ali Mohammadi, Vahid Khaze, Elham Baghbani, Ahad Mokhtarzadeh, Dariush Shanehbandi, Afshin Derakhshani, Pascal H. G. Duijf, Behzad Baradaran

**Affiliations:** 1 Immunology Research Center, Tabriz University of Medical Sciences, Tabriz 5166614766, Iran; b.mansoori_lab@yahoo.com (B.M.); shahgoli@health.sdu.dk (V.K.); e_baghban49@yahoo.com (E.B.); ahad.mokhtarzadeh@gmail.com (A.M.); dariush_shanehbandi@yahoo.com (D.S.); afshin.derakhshani94@gmail.com (A.D.); 2 Student Research Committee, Tabriz University of Medical Sciences, Tabriz 5166614766, Iran; 3 Department of Cancer and Inflammation Research, Institute for Molecular Medicine, University of Southern Denmark, 5000C Odense, Denmark; amohammadi@health.sdu.dk; 4 Medical Oncology Unit-IRCCS IstitutoTumori “Giovanni Paolo II” of Bari, 70124 Bari, Italy; n.silvestris@oncologico.bari.it; 5 Department of Biomedical Sciences and Human Oncology, DIMO-University of Bari, 70124 Bari, Italy; 6 Faculty of Health, Institute of Health and Biomedical Innovation, School of Biomedical Sciences, Queensland University of Technology, 37 Kent Street, Brisbane, QLD 4102, Australia; 7 University of Queensland Diamantina Institute, Translational Research Institute, The University of Queensland, 37 Kent Street, Brisbane, QLD 4102, Australia; 8 Department of Immunology, Faculty of Medicine, Tabriz University of Medical Sciences, Tabriz 5166614766, Iran

**Keywords:** miR-34a, miR-200c, HIF1-α, cancer stemness, apoptosis, cell cycle arrest, metastasis, breast cancer

## Abstract

Breast cancer is the most common women’s malignancy in the world and, for subgroups of patients, treatment outcomes remain poor. Thus, more effective therapeutic strategies are urgently needed. MicroRNAs (miRNAs) have emerged as promising therapeutic tools and targets, as they play significant roles in regulating key cellular processes by suppressing gene expression. However, additive opportunities involving miRNAs have been underexplored. For example, both miR-34a and miR-200c individually suppress the development of different types of cancer, but the cellular effects of their combined actions remain unknown. Here, we show that miR-34a and miR-200c levels are reduced in breast tumors compared to adjacent normal tissues and that this additively predicts poor patient survival. In addition, in cell lines, miR-34a and miR-200c additively induce apoptosis and cell cycle arrest, while also inhibiting proliferation, invasion, migration, stemness and epithelial-to-mesenchymal transition (EMT). Mechanistically, both miRNA-34a and miR-200c directly target HIF1-α and subsequently downregulate VEGFR, MMP9 and CXCR4, although combined miRNA-34a and miR-200c delivery suppresses mouse xenograft tumor development as effectively as individual delivery. We establish a model, supported by in vitro and clinical data, which collectively suggest that the co-delivery of miR-34a and miR-200c represents a promising novel therapeutic strategy for breast cancer patients.

## 1. Background

Human breast cancer (BC) is known as the most common malignancy among women. It is caused by somatic genetic and genomic alterations in breast cells [1,2]. The 5-year survival rate of this disease is about 89.9% [3]. Although recent advances in surgical, chemo-, radio- and hormone-therapy for BC have improved the outcomes, there are still subgroups of BC patients with high morbidity and the underlying causes are not always clear. Some events, such as recurrence, metastasis, and resistance to chemo- and radiotherapy, provide a poor prognosis. In addition, the lack of diagnostic and therapeutic markers for targeted therapy approaches is a major barrier to improving treatment for some patient groups.

MicroRNAs (miRNAs) are molecules of 22 nucleotides in length. They regulate gene expression at the post-transcriptional level by targeting the 3′UTRs of target mRNAs [4]. Accumulating evidence supports that miRNAs are crucial regulatory molecules of carcinogenesis and cancer development and they are increasingly recognized as biomarkers for human cancer diagnosis and prognosis [5]. It has been demonstrated that individual miRNAs can regulate the expression of multiple mRNAs, thereby affecting critical cellular processes, including cell proliferation and differentiation [6]. MiRNAs have also been proposed as novel tools in cancer-targeted therapeutic approaches [7].

According to a recent study, miR-34a expression is reduced in BC, suggesting that aberrant expression of this miRNA could impact BC progression [8]. Moreover, downregulation of this miRNA in several types of malignancies, including BC, suggests that miR-34a acts as a tumor suppressor miRNA [9,10,11]. MiR-34a has also been implicated in senescence, apoptosis, cell cycle progression and epithelial-to-mesenchymal transition (EMT) in cancer stem cells [12,13,14,15]. Increasing evidence has shown that miR-34a targets several important mRNA sequences, including the BCL-2, SIRT1 and NOTCH1 transcripts [16,17]. MiR-34a has also been shown to inhibit EMT by directly targeting MMP9 in tongue cancer [18]. Furthermore, a combination of miR-34a and miR-199-5p might strongly target HIF1-α [19].

The miRNA miR-200c has been established as a biomarker for several types of cancers, such as ovarian cancer [20] and BC [21]. Among the miR-200 family, miR-200c has an important role in the EMT process and drug resistance [22,23]. In addition, it has been identified as a potent inhibitor of tumor development [24]. In lung cancer, miR-200c was shown to target both VEGF and HIF1A [25]. MiR-200c has also been shown to regulate CXCR4 expression, as it targets ZEB1, a key transcriptional regulator which binds to the CXCR4 promoter [26]. However, the role of miR-200c in BC is not yet completely understood.

In this study, we examined the expression levels of miR-34a and miR-200c in BC tissues. In addition, we studied the effects of miR-34a and miR-200c, both individually and in combination, following transfection into BC cells in vitro and co-delivery in a xenograft tumor model in vivo, on the induction of apoptosis and suppression of cell cycle progression, migration, invasion, and cancer stemness. In addition, we explored the underlying molecular mechanisms, culminating in an additive tumor suppression model.

## 2. Methods

### 2.1. Clinical Samples

Twenty-four BC tumor and their adjacent normal tissues were collected from Noor-e-Nejat Hospital (Tabriz, Iran). The samples were snap-frozen and stored at −80 °C. Before sample collection, all patients signed an informed consent letter allowing the researchers to use their samples for research purposes. All materials and protocols used in this study were approved by the Ethics Committee of Tabriz University of Medical Sciences (IR.TBZMED.REC.1395.366).

### 2.2. Bioinformatics Analyses

For microRNA expression analysis related to patient survival, level 3 miRNA HiSeq RNAseq data were downloaded from The Cancer Genome Atlas (TCGA), as previously described [27,28]. Similarly, clinical data were downloaded as described [29]. Patients that were negative for estrogen receptor expression were split into low and high groups, based on whether their tumors’ miR-34a or miR-200c expression levels were lower or higher than the median expression level [30]. To study additives between miR-34a or miR-200c in the context of patient survival, the patients those whose tumors expressed low levels of both miR-34a and miR-200c were compared to those that expressed low levels of only miR-34a or only miR-200c, using the median expression levels as the cut-offs [30]. To assess whether survival curves between the groups significantly differed, Gehan–Breslow–Wilcoxon tests, log-rank tests and Cox proportional hazard analyses were used [31]. An epithelial-to-mesenchymal transition (EMT) score was calculated as previously reported [32], and this score was compared to miR-34a or miR-200c expression levels using Spearman’s rank correlations.

### 2.3. Cell Culture and Transfection

Invasive (MDA-MB-231 and MDA-MB-468), non-invasive (MCF-7, BT and SKBR3) BC and normal breast (MCF-10A) cell lines were obtained from the cell bank of Immunology Research Center (IRC, Tabriz, Iran). Cells were cultured in RPMI-1640 medium containing 10% FBS and 1% Penicillin/Streptomycin (Gibco, Gaithersburg, MD, USA) and incubated at 37 °C with 95% humidity and 5% CO_2_. All experiments were performed with cells in the logarithmic growth phase. For transfections, 1 nmol of mock RNA (negative control), 1 nmol of miR-34a, 1 nmol of miR-200c or the combination of 0.5 nmol of miR-34a and 0.5 nmol of miR-200c were used. Briefly, the mature sequences diluted in 150 µL of NaCl buffer (5 mM) and 5 µL JetPRIME (Polyplus, Illkirch-Graffenstaden, France) transfection reagent were added to each mixture. Then, the whole mixture was added onto the cells drop by drop.

### 2.4. Quantitative Reverse-Transcription PCR

To assess gene expression analysis, total RNA was isolated from cells and tissues using RiboEx reagent (GeneAll, Seoul, Korea) according to the manufacturer’s protocols. Quality and quantity assessment of total RNAs was performed using a NanoDrop2000 spectrophotometer (Thermo Scientific, Waltham, MA, USA). Complementary DNA (cDNA) synthesis was performed via miRCURY LNA Universal RT cDNA Synthesis Kit (Qiagen, Germantown, MD, USA). Briefly, 2 µL of 5 ng RNA template, 2 µL of 5x miRCURY RT Reaction Buffer, 1 µL of 10x miRCURY RT Enzyme Mix, and 5 µL of RNase-free water were added to the microtubes and incubated at 42 °C for 60 min, followed by 95 °C for 5 min in a thermal cycler instrument (Bio-rad, Hercules, CA, USA). The qRT-PCR was performed to detect the expression level of miRNA using the SYBR green method. Briefly, 5 µL master mix (Exiqon, Vedbæk, Denmark), 0.5 µL primer (4 pmol) (Exiqon) (Table 1), 0.5 µL cDNA template, and 4 µL distilled H_2_O (dH_2_O) were added to the 0.1 strips (Gunster, New Taipei City, Taiwan). The strips were incubated according to the following thermal cycling protocol: 95 °C for 10 min, 45 cycles, including 95 °C for 10 s, and 60 °C for 60 s using a light cycler system (Roche, Mannheim, Germany).

### 2.5. Apoptosis Assays

Apoptosis assays were performed using the Annexin V-FITC Apoptosis Detection Kit (EXBIO, Vestec, Czech Republic). According to the Exbio protocol, cells were harvested and washed using phosphate buffer; then, 100 µL binding buffer, 5 µL annexinV, and 5 µL propidium iodide (PI) were added to each sample and incubated for 20 min in the dark. The cells were centrifuged, and cell pellets were re-suspended in binding buffer. Finally, the cells were analyzed by a MACSQuant 10 flow cytometer instrument (Miltenyi biotec, Bergisch Gladbach, Germany). Flow cytometry data were analyzed using FlowJO software (FlowJo LLC., Ashland, OR, USA).

### 2.6. Cell Cycle Profiling

Different phases of the cell cycle were detected using DAPI dye and analyzed using flow cytometry. Briefly, the cells were harvested and washed with phosphate buffer, fixed with 75% ethanol and incubated at −20 °C overnight. Next, the cells were washed and incubated in phosphate buffer containing 1% RNase A in cell culture condition for 30 min. Then, the cells were washed and stained with DAPI solution (0.1% DAPI (1 mg/mL), 0.1% Triton X100 in PBS) for 10 min in RT and dark conditions. Finally, the cells were analyzed using the MACSQuant 10 flow cytometer instrument (Miltenyi biotec). Different phases of the cell cycle were analyzed by FlowJO software (FlowJo LLC.).

### 2.7. Invasion Assays

Invasion assays were performed by transwell chambers coated with matrigel. Briefly, 5 × 10^3^ cells were re-suspended in RPMI-1640 without FBS and seeded in the transwell chambers; additionally, RPMI-1640 containing 20% FBS was added to the bottom chamber. The membrane was stained with Giemsa. After clearing the surface of the membrane, the cells that invaded through the 8 µm pores were counted under an inverted light microscope (Optica, Ponteranica, Italy).

### 2.8. Migration Assays

BC cell migration was evaluated using scratch assays. A total of 1×10^5^ cells were seeded in 24-well cell culture plates. The scratch was applied to untreated and transfected with miR-34a, miR-200c, and miR-34a + miR-200c wells. The cells that migrated to the “wound” area were counted after 48h using ImageJ software (NIH, Bethesda, MD, USA).

### 2.9. Proliferation Assays

BC cell proliferation was performed using MTT assays. A total of 15 × 10^3^ cells were seeded in 96-well cell culture plates. After transfection, 50 µL of MTT (2 mg/mL) was added to each well containing RPMI-1640 with 10% FBS. Next, the cells were incubated for 4 h in a cell culture incubator. After that, formazan crystals were dissolved by adding 100 µL of dimethyl sulfoxide and shaking in 100 rpm. Finally, the optical density was measured in 570 nm using a microplate reader (TECAN, Sunrise, Grödig, Austria).

### 2.10. Western Blot Analysis

Total protein was extracted from transfected cells using RIPA lysis buffer (Santa Cruz Biotechnology, Santa Cruz, CA, USA) according to the manufacturer’s protocol. A total of 50 µg of total protein was loaded into each well of SDS-PAGE gels with 4% stacking and 10% running buffer. Then, the proteins were blotted onto the PVDF membrane and blocked with blocking buffer (0.5% Tween-20 in PBS). Then, the membrane was incubated with primary mouse monoclonal antibody against CXCR4, VEGFR, MMP9, and HIF1-α (1:1000) (Santa Cruz Biotechnology, Santa Cruz) overnight at 4 °C. Next, the membrane was incubated with secondary goat anti-mouse antibody conjugated with horseradish peroxidase (HRP) on a shaker for 1 h. The protein bands were visualized by ECL reagent (Roche) using a Western blot imaging system (Sabz Co., Tehran, Iran).

### 2.11. Colony Formation Assays

A total of 1000 cells were seeded per well in 6-well cell culture plates and incubated to enable colony formation. When the colonies reached more than 25 cells, the cells were transfected with miR-34a, miR-200c, and miR-34a + miR-200c. Subsequently, the wells were stained with crystal violet staining dye for 40 min and the number of colonies was counted in each group.

### 2.12. CD44 and CD133 Evaluation Assays

To understand the effect of miR-34a, miR-200c, and miR-34a + 200c on cancer stemness markers, such as CD44 and CD133, flow cytometry assays were performed on these surface markers. Briefly, the cells were harvested and washed with FACS buffer (PBS, 10% FBS, and 0.1% sodium azide). Then, the cells were treated with primary CD44 and CD133 antibodies, and the cells were incubated with secondary anti-mouse conjugated with FITC. Finally, FITC-positive cells were measured by Macs quant10 flow cytometry instrument and the data analyzed by FlowJo software (version 7.6).

### 2.13. Animal Experiments

A total of 20 athymic female nude mice (nu/nu) (6–8 weeks of age) were obtained from the Australian Animal Resource Centre (ARC, Canning Vale, Australia). A total of 1 × 10^6^ MDA-MB-231 cells which stably expressed luciferase, were subcutaneously injected into the flank. When the tumor grew to a 5 mm diameter, the mice were divided into four groups of mock, miR-34a, miR200c, and miR-34a + miR-200c. Before treatment, the miRNA mature sequence was prepared to inject in an intra-tumoral manner using in vivo-jetPEI^®^-Gal reagent (Polyplus, Illkirch-Graffenstaden, France). Briefly, in one microtube, a total of 5 µg of miRNA was diluted in 10% glucose in ddH_2_O, and in the other microtube, 0.8 µL in vivo-jetPEI^®^-Gal (N/P = 8) was diluted in 10% glucose in ddH_2_O. Then, the tubes were mixed and incubated for 15 min at room temperature (RT). Finally, the reagent contained specific sequences, which were injected intra-tumorally. Tumors were harvested, weighed, and their volumes were measured and analyzed. The experimental procedures were approved by the Health Sciences Animal Ethics Committee (University of Queensland, Brisbane, Australia; approval number UQDI/TRI/433/14/ECF).

### 2.14. In Vivo Colonization and Metastasis Assays

Colonization and metastasis of MDA-MB-231 cells to a distant site of the animal body was evaluated and analyzed using the IVIS Imaging System (Perkin Elmer, Waltham, MA, USA) in the Preclinical Imaging Facility of the Translational Research Institute (TRI, Brisbane, Australia).

### 2.15. Immunohistochemistry

Tissues were fixed with 10% formaldehyde and processed and embedded in paraffin. Then, the paraffin blocks were cut into 5-µm-thick sections. Immunohistochemistry (IHC) was performed after deparaffinization and rehydration. Antigen retrieval was performed using sodium citrate at 100 °C. After that, the slides were incubated with primary mouse antibodies against Ki-67, VEGFR, MMP9, HIF1-α or CXCR4 (1:500) overnight at 4 °C in a humidity chamber. Secondary HRP-conjugated goat anti-mouse antibody (1:200) was added and allowed to bind for 1 h. Protein levels were detected using 3, 30-diaminobenzidine tetrahydrochloride (DAB) substrate. Finally, the sections were stained with hematoxylin and eosin (H&E).

### 2.16. TUNEL Assays

TUNEL assays were performed on sections extracted from animal tumors. TUNEL assays were performed using TUNEL Chromogenic Apoptosis Detection Kit (GeneCopoeia, Rockville, MD, USA) according to GeneCopoeia instructions. Briefly, after the deparaffinization and rehydration of sections, samples were incubated with 2% hydrogen peroxide and the sections were incubated with 50 μL of TdT reaction cocktail for 60 min at 37 °C. Next, the reaction was stopped by adding stop solution (300 mM NaCl and 30 mM sodium citrate). Subsequently, 100 μL of HRP-Streptavidin staining solution was added on the sections and incubated for 1 h at RT. Finally, 100 µL of DAB solution was added to each section and the section was evaluated after staining with H&E.

### 2.17. Statistical Analyses

The data were expressed as mean ± SD. The expression levels of miR-34a and miR-200c in the tumors were compared to those in adjacent normal tissues using non-parametric Mann–Whitney *U* tests. Sample sizes in animal experiments were chosen on the basis of the literature documentation according to the equation $n\;=\;1\;+\;2C{\left.\left(\frac{S}{D}\right.\right)}^{2}$. Herein, *n* is the number of animals, *C* is a constant based on power and significance, *s* is the standard deviation and *D* is the group difference. Correlations between miR-34a and miR-200c expression were analyzed by Pearson’s correlation coefficient. A *p*-value less than 0.05 was considered statistically significant for each group compared to the negative control (NCTRL) group. All experiments and assays were performed in triplicate.

## 3. Results

### 3.1. MiR-34a and miR-200c Are Downregulated in Breast Cancer Tissues and Invasive Cell Lines and This Predicts Poor Patient Survival

**Figure 1:**
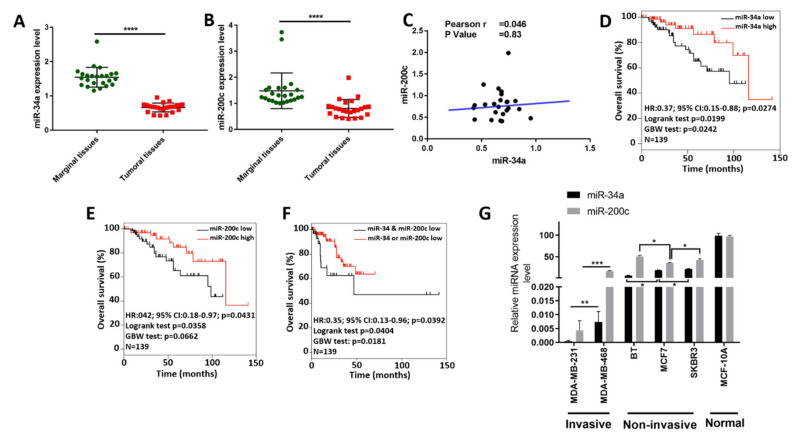
Downregulation of miR-34a and miR-200c is associated with breast cancer development. (**A**,**B**) Expression levels of miR-34a (**A**) and miR-200c (**B**) in breast cancer (**B**,**C**) and adjacent normal tissues, as determined by qRT-PCR. ****, *p*-value < 0.0001. (**C**) Pearson correlation analysis between miR-34a and miR-200c expression levels. (**D**,**E**) Overall survival curves for ER- breast cancer patients comparing patients whose tumors express high and low levels of miR-34a and miR-200c, respectively, using the median expression as the cut-off. HR: Hazard ratio; CI: confidence interval; GBW: Gehan-Breslow–Wilcoxon. (**F**) Overall survival curves as in (**D**,**E**), but here comparing patients whose tumors express both low miR-34a and low miR-200c to those expressing either low miR-34a or low miR-200c (using median cut-offs). (**G**) Expression levels of miR-34a and miR-200c in different invasive (MDA-MB-231 and MDA-MB-468), non-invasive BC cell lines (BT, MCF-7 and SKBR3) and normal breast cell line (MCF-10A), as determined by qRT-PCR. Abbreviations: *, *p*-value < 0.05; **, *p*-value < 0.01; ***, *p*-value < 0.001.

We analyzed the expression of miR-34a and miR-200c in BC tissues and adjacent normal breast tissues by qRT-PCR. The expression level of miR-34a was significantly downregulated in the tumor tissues compared to adjacent normal tissues (Figure 1A). In addition, miR-200c levels were decreased in breast tumors compared to the normal tissues (Figure 1B). Pearson correlation analysis did not show a significant correlation between the expression of these two miRNAs (Figure 1C).

We also found that reduced miR-34a and miR-200c expression are significantly associated with poor BC patient survival in more malignant types of BC, which are negative for estrogen receptor expression (*p* < 0.05, as per both log-rank and Cox proportional hazard tests for each miRNA) (Figure 1D,E). In addition, patients whose tumors simultaneously express both low miR-34a and low miR-200c show a significantly poorer overall survival rate than patients whose tumors express either low miR-34a or low miR-200c (*p* < 0.05, as per both log-rank and Cox proportional hazard tests) (Figure 1F). This suggests that low miR-34a and low miR-200c expression do not only individually forecast poor prognosis, but they also predict poor survival outcome.

Among the five BC cell lines, miR-34a was significantly decreased in both invasive BC cell lines (*p* < 0.01). In addition, miR-200c expression was significantly lower in MDA-MB-231 than in MDA-MB-468 cells (*p* < 0.001). Among the non-invasive cell lines, BT showed the lowest miR-34a expression (*p* < 0.05) and MCF-7 showed the lowest miR-200c expression (*p* < 0.05). The expression level of both miR-34a and miR-200c in normal breast cell line was significantly higher than invasive and noninvasive BC cell lines (*p* < 0.0001) (Figure 1G).

Taken together, these results indicate that miR-34a and miR-200c are downregulated in breast cancer and invasive breast cancer cell lines, and that they are additionally associated with poor patient survival.

### 3.2. Restoration of miR-200c and miR-34a Expression in Breast Cancer Cell Lines Additionally Induces Apoptosis and G_2_/M Cell Cycle Arrest

**Figure 2:**
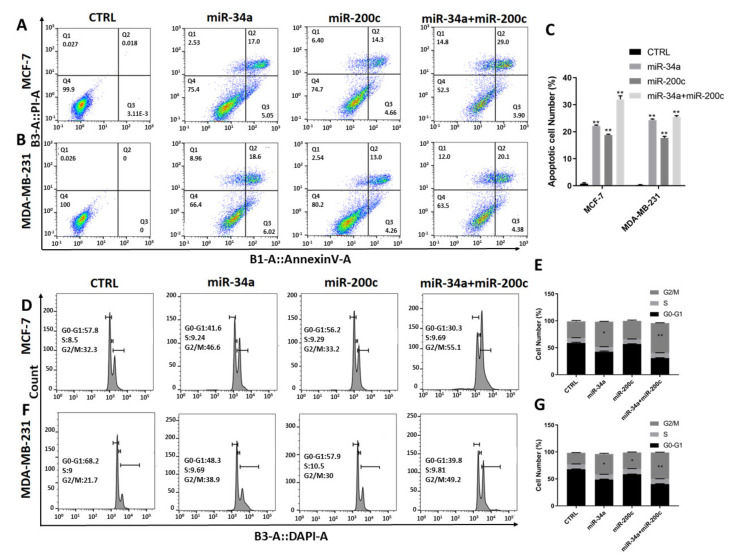
Exogenous miR-34a and miR-200c expression additionally induce apoptosis and G_2_/M cell cycle arrest. (**A**–**C**) Annexin V/propidium iodide assays following transfection of miR-34a, miR-200c and the combination in MCF-7 and MDA-MB-231 breast cancer cell lines. (**D**,**E**) Cell cycle profiles and respective quantifications of MCF-7, and (**F**,**G**) and MDA-MB-231 cells stained with DAPI and subjected to flow cytometry. Abbreviations: *, *p*-value < 0.05; **, *p*-value < 0.01.

Given the downregulation of miR-34a and miR-200c in breast tumors and cell lines, we next studied the effect of restoration of miR-34a and miR-200c expression, via transfection, on apoptosis induction using annexin V/PI assays. Based on the above results, we chose to use the non-invasive MCF-7 and the invasive MDA-MB-231 cell lines for these experiments. The results show that while mock transfection does not induce apoptosis, miR-34a induces apoptosis in 22.2 ± 0.28% and 24.35 ± 0.35%, miR-200c induces apoptosis in 18.8 ± 0.28% and 17.68 ± 0.59%, and the combination induces apoptosis in 32 ± 1.27% and 25.45 ± 0.49% of MCF-7 and MDA-MB-231 cells, respectively (Figure 2A,B).

Additionally, we performed cell cycle assays using DAPI staining and flow cytometry. The results show increased cell populations in the G_2_/M phase of the cell cycle in both MCF7 and MDA-MB-231 cells. Specifically, the rates are 47.1 ± 0.70 and 38.05 ± 1.2 percent for miR-34a, 34.2 ± 1.4 and 30.6 ± 0.84 percent for miR-200c and 55.6 ± 0.70 and 48.9 ± 0.42 percent for miR-34a and miR-200c co-transfection using MCF7 and MDA-MB-231 cells, respectively (Figure 2C,D).

Thus, increased expression of miR-34a and miR-200c in these breast cancer cell lines induces apoptosis and G_2_/M cell cycle arrest, and this effect is exacerbated by combining exogenous miR-34a and miR-200c expression. Importantly, the total amount of transfected miRNA is constant in each group (see Methods). Therefore, miR-34a and miR-200c additionally induce apoptosis and G_2_/M cell cycle arrest.

### 3.3. MiR-200c and miR-34a Additionally Inhibit Breast Cancer Cell Invasion, Migration, Proliferation and Expression of EMT Markers

**Figure 3:**
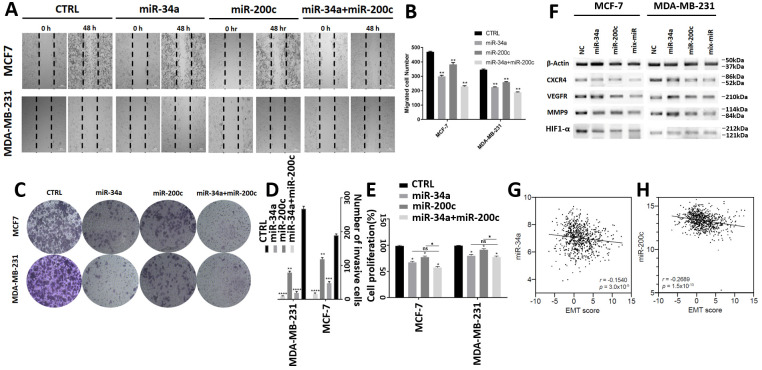
MiR-34a and miR200c additionally suppress breast cancer cell migration, invasion, proliferation and expression of markers for the epithelial-to-mesenchymal transition. (**A**) Light microscope images showing the migration of indicated cells in scratch “wound” assays. Scratch areas are marked by hatched lines. Images show timepoints 0 and 48 h (h) after application of the scratches. Cells were transfected with indicated miRNAs. (**B**) Quantification of the numbers of cells that migrated into the scratch areas after 48 h, as shown in (**A**). **, *p*-value < 0.01. (**C**) Images of invasion assay plates of transfected breast cancer cell lines, as indicated. (**D**) Quantification of cell invasion in transfected cells, as shown in (**C**). **, *p*-value < 0.01; ***, *p*-value < 0.001; ****, *p*-value < 0.0001. (**E**) Quantification of BC cell proliferation relative to control transfected cells. (**F**) Western blots showing changes in expression levels of EMT-promoting proteins after miRNA transfection. (**G**,**H**) Correlations between an EMT score and miR-34a or miR200c expression, respectively, in breast tumors (each *n* = 729). Data are derived from TCGA and show the Spearman’s rank correlation coefficients and *p* values. Abbreviations: CTRL: control; *, *p*-value < 0.05.

To assess whether miR-34a, miR-200c and the combination affect BC cell migration and invasion, we performed wound healing and Matrigel trans-well invasion assays. This showed that individual transfection of miR-34a and miR-200c reduces both BC cell migration and invasion. Similar to the above observations, co-transfection additionally decreases cell migration and invasion (Figure 3A–D).

Next, we performed proliferation assays. This also showed an additive decrease in BC cell proliferation in miR-34a and miR-200c co-transfected cells compared to cells transfected with miR-34a or miR-200c individually, which also reduces cell proliferation (Figure 3E). However, we note that the additive effect is not significant for the co-transfected group compared to the miR-34a treated group in both cell lines.

In addition, we performed Western blot analyses to assess how miR-34a and miR-200c may affect the expression levels of proteins involved in EMT, a key malignant cellular process that precedes metastasis. This demonstrates that miR-34a and miR-200c typically decrease the expression of proteins that promote EMT, including CXCR4, VEGFR, MMP9 and HIF1-α, and the miR-34a and miR-200c combination shows an additive reduction (Figure 3F).

We also assessed miR-34a and miR-200c expression in breast cancer samples from TCGA and compared them to an EMT score, as previously reported [32]. This showed that both miR-34a and miR-200c expression negatively correlate with EMT (each Spearman *p* < 0.0001) (Figure 3G,H). Thus, consistent with our in vitro results, increased expression levels of miR-34a and miR-200c are also associated with reduced EMT in breast cancers.

Taken together, our experiments show that miR-34a and miR-200c additionally suppress breast cancer cell migration, invasion, proliferation and the expression of proteins that promote EMT.

### 3.4. MiR-34a and miR-200c Additionally Decrease Breast Cancer Stemness Properties

**Figure 4:**
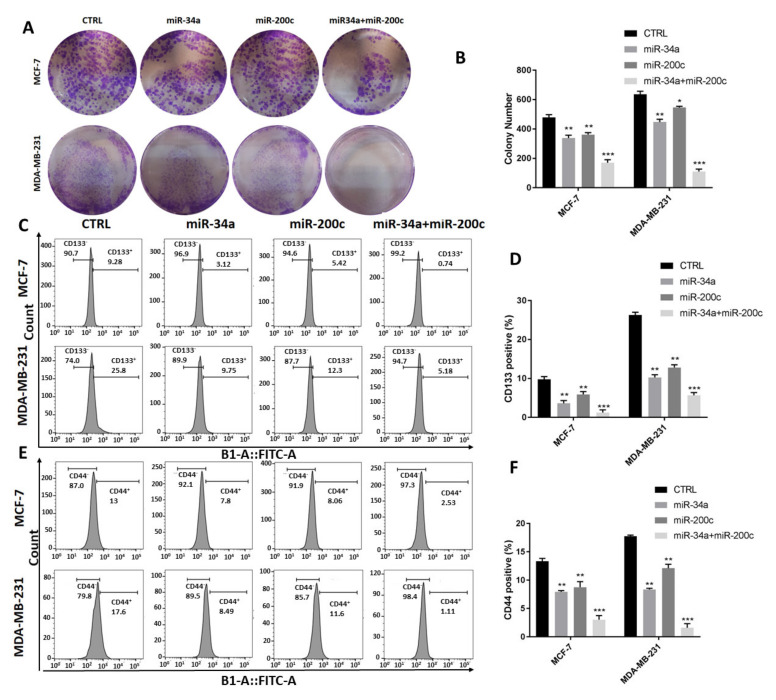
MiR-34a and miR-200c additionally inhibit stemness features of breast cancer cells. (**A**) Representative images of colony formation plates pertaining to indicated cell lines transfected with indicated miRNAs. (**B**) Quantification of colony numbers from experiments shown in (A). *, *p*-value < 0.05; **, *p*-value < 0.01; ***, *p*-value < 0.001. (**C**–**F**) Flow cytometry of indicated cells stained for cancer stem cell markers CD133 (**C**,**D**) and CD44 (**E**,**F**). Histograms are shown in (**C**,**E**). Quantification of CD133^+^ and CD44^+^ cells are shown in (**D**,**F**). **, *p*-value < 0.01; ***, *p*-value < 0.001.

We next study how miR-34a and miR-200c might affect cancer stem cell properties in the context of breast cancer development. To that end, we performed colony formation assays. This revealed that combined miR-34a and miR-200c expression decreases the number of colonies formed by co-transfected BC cells more significantly than those formed by cells that were transfected with miR-34a or miR-200c individually, and this applies to both invasive and non-invasive cells (Figure 4A,B).

We also investigated the effects of miR-34a or miR-200c on stemness by determining the expression levels of stem cell markers, including CD133 and CD44, as we evaluated the populations of CD133^+^ and CD44^+^ cells using flow cytometry. Following co-transfection, the percentages of CD133^+^ cells are significantly decreased compared to cells transfected with the two miRNAs individually (Figure 4C,D). Similarly, combined miR-34a and miR-200c transfection reduce the fraction of CD44^+^ cells more profoundly compared to single transfections (Figure 4E,F).

Thus, we conclude that miR-34a and miR-200c additionally decrease breast cancer stemness properties.

### 3.5. MiR-34a and miR-200c Inhibit Cancer Development In Vivo

**Figure 5:**
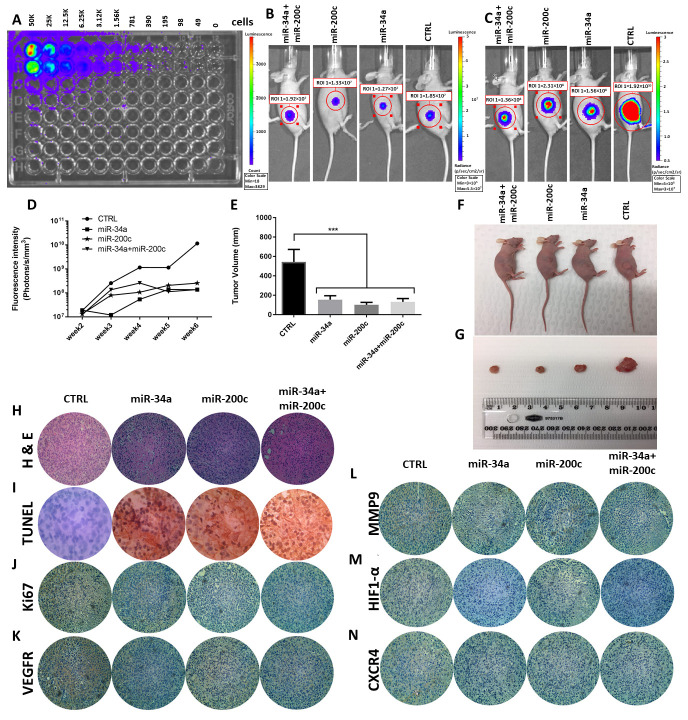
Individual and co-delivery of miR-34a and mir-200c suppress cancer development in a xenograft mouse model. (**A**) Bioluminescence imaging of serially diluted MDA-MB-231 cells, which stably express luciferase. (**B**) Bioluminescence imaging of mice with tumor formation prior to miRNA treatment. (**C**) Bioluminescence imaging of mice after intra-tumor injections with indicated miRNAs. (**D**) Quantification of bioluminescence intensities of mouse tumors after intra-tumor injections with indicated miRNAs. Measurements of tumor volumes (**E**), Images of mice (**F**), excised tumors (**G**) for indicated groups. ***, *p* < 0.001. (**H**,**I**) H&E staining on each tumor (**H**) and TUNEL assay to show apoptosis induction (**I**) after miRNA transfection. (**J**–**N**) Immunohistochemistry staining for Ki67, VEGFR, MMP9, HIF1-α and CXCR4, respectively.

To examine the importance of miR-34a and miR-200c in tumor development, we performed in vivo xenograft mouse modelling. For this, we used MDA-MB-231 cells that stably express luciferase. First, we serially diluted these cells in vitro to demonstrate the suitability of these cells for quantitative detection by bioluminescence imaging (Figure 5A). Next, we injected these cells into mice and allowed xenograft tumors to form of up to 5mm in diameter (Figure 5B). Then, we delivered mature miRNAs, specifically control, miR-34a, miR-200c and the combination, to the tumors via intra-tumor injections and monitored further tumor development over time. The fluorescence density of the tumors was measured once a week, from the second up to the fifth week. Control tumors continued to develop rapidly, as evidenced by a significant increase in tumor fluorescence intensity, whereas the tumors in the treated groups developed more slowly (Figure 5C,D). In the co-treatment miR-34a and miR-200c group, the fluorescence intensity was similar to those in the individual miR-34a and mir-200c groups (Figure 5C,D). Similarly, the tumor volumes were significantly decreased in all miRNA-treated groups compared to the negative control group (Figure 5E–G). TUNEL assays on the mouse tumors showed TUNEL-positive cells in the three miRNA-treated groups but not in the control group, suggesting increased rates of apoptosis in response to the miRNAs (Figure 5I). In addition, immunohistochemistry on tumor sections from the xenograft models showed that both individual treatment and co-treatment decreases the protein expression levels of Ki-67, VEGFR, MMP9, HIF1-α and CXCR4 compared to control tumors (Figure 5J–N). These results indicate that the delivery of miR-34a and miR-200c, both individually and in combination, inhibits cancer development in vivo. However, the additive effect between these miRNAs that we observed in vitro does not seem to be sustained in vivo.

## 4. Discussion

**Figure 6:**
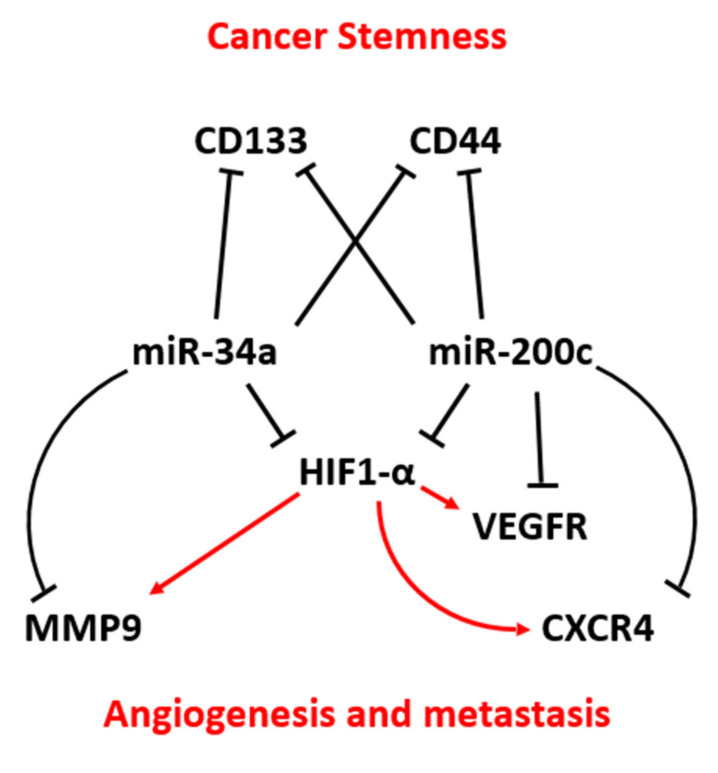
A model explaining how miR-34a and miR-200c may cooperate by targeting unique and shared components of key signaling pathways to jointly suppress angiogenesis, metastasis and cancer stemness features in breast cancer.

In this study, we first evaluated the expression levels of miR-34a and miR-200c in clinical BC samples. This showed a significant downregulation of miR-34a and miR-200c in BC compared to adjacent normal breast tissue. Pearson correlation analysis did not show a significant correlation between miR-34a and miR-200c expression in these samples. However, we found that reduced expression of both miR-34a and miR-200c is associated with poor prognosis for breast cancer patients with estrogen-receptor-negative BC, a more malignant type of BC. We also observed that the co-transfection of miR-34a and miR-200c could promote apoptosis and G_2_/M cell cycle arrest and inhibit a broad range of malignant cellular features characteristic of breast cancer development, including the proliferation, invasion, migration and expression of key markers of EMT and stemness (summarized in Figure 6). There is some previous evidence suggesting that miR-34a and miR-200c individually act as tumor suppressors. For example, Li et al. demonstrated that of miR-34a is downregulated in BC tumor tissues and cell lines [16]. In another study, Song et al. showed that miR-200c is downregulated in eight different BC cell lines and tumors [33]. However, the expression profiles and potential combined action of miR-34a and miR-200c—and hence therapeutic perspective—were still unclear. The main novelty of our current study lies in the identification of additives between miR-34a and miR-200c. Indeed, these miRNAs act as additive tumor suppressors in the context of seven out of seven abovementioned tumor-suppressive actions, ranging from promoting apoptosis to reducing stemness features, and we observe additive effects in the context of breast cancer patient survival outcome (see below).

A large body of studies is consistent with our data in the context of breast cancer and other cancer types, showing that mir-34a and miR-200c individually significantly suppress cancer development and cell proliferation [34,35]. The transactivation of miR-34a suppresses cell proliferation and induces apoptosis by induction of the p53 pathway [36]. In addition, Song et al. showed that miR-34a replacement increases the sensitivity to cisplatin by inducing apoptosis in lung cancer [37]. Swartling et al. established that miR-34a induces apoptosis and G_2_ arrest in medulloblastoma and potentially increases the sensitivity of these cells to chemotherapeutic agents [38]. Besides, it was shown that miR-200c regulates apoptosis in endometrial carcinoma cells [39]. In addition, miR-200c overexpression significantly induces apoptosis in lung cancer [34]. Likewise, treatment with miRNA-200c promotes G_2_ arrest in esophageal cancer cells [40]. In another experiment, it was shown that miRNA-200c promotes G_2_/M and G_1_ arrest caused by the downregulation of cyclin B1 and CDK1 and upregulation of p21 [41]. Inconsistent with our results, some studies report that both miR-34a and miR-200c could induce G_1_ arrest [42,43]. We note that cell cycle arrest and apoptosis may be linked. A process referred to as “mitotic catastrophe” may result in apoptosis following mitotic arrest [44]. In addition, p53 status dictates the ability and efficacy of cells to invoke cell cycle arrest, whether in G_1_, G_2_ or M phase and apoptosis [45]. Thus, it is possible that seemingly inconsistent observations in the literature can be explained by the p53 status of the cell lines that were used [46].

We found that inducing miR-34a and miR-200c together suppresses cancer stem cell features more so than single miRNAs transfection, as observed by the reduced formation of BC colony numbers and decreased expression of the cancer stem cell markers CD44 and CD133. Recent evidence showed that miR-34a targets both CD44 [13] and CD133 [47,48] and this resulted in the suppression of cancer stem cell features, such as self-renewal capacity, in various cancer cells. Moreover, miR-200c inhibits CD44 and increases the sensitivity of glioma cells to chemotherapeutic agents [23]. Generally, miR-200c suppresses CD44 by targeting *FUT4* and α1.3-fucosylation involved in the formation of CD44 [41]. Thus, this adds to our model (Figure 6).

Our results also show that in combination, miR-34a and miR-200c inhibit BC invasion and migration more effectively in vitro. For example, the protein expression levels of VEGFR, MMP9, HIF1-α, and CXCR4 significantly decrease after the transfection of single miRNAs treatment, and this effect is exacerbated by combined treatment in vitro. In addition, the Ki-67 proliferation marker was significantly decreased after co-treatment, indicating that miR-34a and miR-200c together additionally suppress BC proliferation. Consistently, there is evidence showing that miR-34a antagomirs promote metastasis in CD44^-^ prostate cancer cells, suggesting that downregulation of miR-34a in cancer stem cells contributes to metastasis via regulation of CD44 expression [49]. MiR-34a was also found to inhibit invasion and migration by targeting MMP9 in tongue squamous cell carcinoma [50]. In addition, it was demonstrated that miR-34a, in combination with other miRNA, including miR-199a and miR-20b, could target HIF-1α [51,52]. In another study, Shan et al. showed that downregulation of miR-200c might increase tumor metastasis via EZH2/E-cad pathway in lung malignancy [34]. We have previously shown that miR-200c targets VEGFR and MMP9 in gastric cancer cell lines [53] and miR-200c inhibits CXCR4 indirectly by targeting ZEB1 [54]. Additionally, miR-200c inhibits HIF-1α and VEGF expression in U251 and A549 cancer cells [25]. In yet another study, both mRNA and protein levels of HIF-1α were shown to be reduced after miR-200c transfection in hypoxic lung cancer [55]. Together, these studies suggest a main role of miR-200c in invasion, migration, and angiogenesis in BC. This is consistent with our model, in which miR-34a and miR-200c act in parallel and their actions converge to activate the same tumor-suppressive pathways (Figure 6). Additional cross-talk within these pathways has been observed. For example, HIF-1α increases the expression level of VEGFR [56] and it binds to the promoter of MMP9 [57] and CXCR4 [58] to increase the expression levels of these proteins. Thus, integrating this knowledge into our model, miR-34a and miR-200c have common targets (i.e., HIF-1α) and individual targets (e.g., MMP9, VEGFR), which, together, may decrease BC cell invasion, migration and angiogenesis (Figure 6).

Importantly, while we identified profound additive tumor-suppressive effects of miR-34a and miR-200c in vitro, we did not observe additive tumor suppression in our mouse xenograft model. Individual delivery of miR-34a and miR-200c to tumors suppresses their development as effectively as combined delivery, as observed by both bioluminescence and tumor size measurements. Consistently, compared to those in control tumors, the levels of apoptosis and expression of EMT and stemness markers are all significantly different in tumors treated with miR-34a and miR-200c individually and in combination, but combination therapy is no more effective that individual therapy. Despite this, we argue that combination treatment, using miR-34a and miR-200c together, rather than each one alone, constitutes a stronger potential therapeutic approach. This argument is based upon the following. First, we observe strong additive tumor-suppressive effects in vitro, involving the additive suppression of several cancer hallmark features, including proliferation, invasion, migration, EMT and stemness, as well as key additive tumor suppressive actions, such as increased apoptosis and increased G_2_/M cell cycle arrest. Second, in patients, we observe an additive survival effect, because patients with low expression levels of both miR-34a and miR-200c show significantly poorer survival outcome than patients with low expression levels of only miR-34a or only miR-200c. This demonstrates clear relevance for breast cancer patients, irrespective of our observations using mouse xenograft modeling. Third, our in vitro observations are consistent with a large body of the literature, showing that miR-34a and miR-200c contribute to tumor suppression by invoking the same, as well as parallel, pathways (Figure 6). This is important, because if tumors have acquired mutations—maybe inherently in the tumor or after drug treatment—then triggering the same tumor-suppressive pathways from multiple different angles, here miR-34a and miR-200c together, is likely to be a far more effective therapeutic strategy.

In conclusion, we found that miR-34a and miR-200c are downregulated in BC tissues, identifying miR-34a and miR-200c as diagnostic biomarkers for BC. The combination of miR-34a and miR-200c additionally induces apoptosis and G_2_/M cell cycle arrest and inhibits the proliferation, invasion, migration and cancer stemness properties of BC cells. Although we did not observe additive tumor suppression in a mouse xenograft model, we provide several arguments that support our proposal that dual treatment with miR-34a and miR-200c could represent a novel effective therapeutic approach to treat BC.

## Figures and Tables

**Table 1:** Primer sequences.

	Target Sequence (5′-3′)
miR-34a	UGGCAGUGUCUUAGCUGGUUGU
miR-200c	CGUCUUACCCAGCAGUGUUUGG

## Data Availability

The data that support the findings of this study are available from B.B. but restrictions apply to the availability of these data, which were used under license for the current study, and so are not publicly available. Data are however available from the authors upon reasonable request and with permission of B.B.
